# Cardiovascular Disease Risk Factors: How Relevant in African Men With Prostate Cancer Receiving Androgen-Deprivation Therapy?

**DOI:** 10.1200/JGO.2015.002790

**Published:** 2016-05-25

**Authors:** Okon Ekwere Essien, Iya Eze Bassey, Rebecca Mtaku Gali, Alphonsus Ekpe Udoh, Uwem Okon Akpan, Enakirerhi E. Glen

**Affiliations:** **Okon Ekwere Essien**, **Iya Eze Bassey**, **Alphonsus Ekpe Udoh**, and **Uwem Okon Akpan**, University of Calabar College of Medical Sciences; **Enakirerhi E. Glen**, University of Calabar Teaching Hospital, Calabar; and **Rebecca Mtaku Gali**, University of Maiduguri College of Medical Sciences, Maiduguri, Nigeria.

## Abstract

**Purpose:**

Cardiovascular disease risk factors have been associated with androgen-deprivation therapy (ADT) in white and Hispanic populations. It is therefore relevant to determine if there exists a relationship between these parameters in the African population.

**Patients and Methods:**

The design of the study was cross sectional. Prostate-specific antigen concentration, waist circumference, body mass index (BMI), lipid profile, glucose level, and insulin level were determined in 153 patients with prostate cancer and 80 controls. The patients with prostate cancer were divided into subgroups of treatment-naïve patients and those receiving ADT.

**Results:**

Mean total cholesterol (*P* = .010), LDL cholesterol (*P* = .021), BMI (*P* = .001), and waist circumference (*P* = .029) values were significantly higher in patients treated with ADT when compared with treatment-naïve patients. In patients treated with ADT for up to 1 year, only mean BMI was significantly higher than in treatment-naïve patients, whereas those treated with ADT for more than 1 year had significantly higher mean BMI, waist circumference, total cholesterol, and LDL cholesterol values when compared with treatment-naïve patients. There were no significant differences in insulin or glucose levels. Those undergoing hormone manipulation after orchiectomy had fewer cardiovascular risk factors compared with those undergoing hormone manipulation alone.

**Conclusion:**

This study shows that ADT results in elevated total cholesterol, LDL cholesterol, BMI, and waist circumference values, all of which are risk factors of cardiovascular disease. Screening for cardiovascular risk factors should be included in treatment plans for patients with prostate cancer.

## INTRODUCTION

The most commonly diagnosed cancer and the second leading cause of cancer death in men globally is prostate cancer (CaP).^1,2^ Currently, CaP is now the leading cancer in men of black African ancestry.^3^ It has been reported that the risk of having this cancer is approximately 60% higher among African American men compared with whites. When compared with any other racial or ethnic group in the United States, African American men have the highest mortality rate. It is more than two times higher than that of white men.^4^

In West Africa, reported incidence of CaP varies from 14 cases per 100,000 men in Gambia and Cape Verde to 6,236 cases per 100,000 men in Nigeria.^5^ In Nigeria, studies in Jos, Sokoto, and Ibadan conducted between 1985 and 1994 reported that CaP constituted 5.7% of recorded cancer cases. In similar studies conducted between 1995 and 2002, the proportion of CaP cases increased to 7.9%, raising it to third place among recorded cancer cases.^6^ Incidence is still on the rise. Abdulkareem et al^6^ reported that CaP was the most common cancer in Nigerian men, accounting for 6.1% to 19.5% of all cancers, and that incidence was increasing. The average prevalence in Nigeria is reported to be 11%. Records from most parts of Nigeria show it to be the third most common cancer, except in Calabar, where a high figure was recorded for CaP as the most common cancer in both sexes. It accounted for 34.7% of all hospital-recorded cases of cancer and 80% of hospital-recorded cases of cancer in men.^6^

Sex hormones are one of the driving forces in the pathogenesis of CaP, which is why treatment of this disease is often based on hormone-manipulation or -modification therapy,^7^ otherwise known as androgen-suppression therapy or androgen-deprivation therapy (ADT). Androgen deprivation is achieved using gonadotropin-releasing hormone (GnRH) agonists, steroidal and nonsteroidal antiandrogens, estrogens, or bilateral orchiectomy.^8^ The aim of ADT is to reduce serum testosterone levels to less than 0.5 mg/L as recommended by current guidelines. The normal range for testosterone in young men is 3 to 10 mg/L.^9^ However, many adverse effects have been associated with male hypogonadism (regardless of etiology). They include impotence, diminished libido, reduced muscle strength and lean body mass, increased fat mass, diminished quality of life, and osteoporosis.^10,11^ Studies have reported increases in hyperlipidemia, obesity, and hyperglycemia as a consequence of ADT in patients with CaP.^11-14^ All these are cardiovascular risk factors and may result in a double burden of disease in patients with CaP. Most of these research studies were conducted in white, Hispanic, and Japanese populations; we wondered if similar observations would be found in African patients with CaP undergoing ADT, who would have different diet, environmental, lifestyle, and genetic factors compared with previous study populations. Our study also examined the effects of therapy with orchiectomy and hormone manipulation alone on risk factors of cardiovascular disease (CVD) in patients with CaP.

## PATIENTS AND METHODS

### Study Design and Patient Selection

The design of the study was cross sectional. One hundred fifty-three patients of Nigerian origin with confirmed CaP attending the Surgical Out Patient Clinic of the University of Calabar Teaching Hospital were recruited as participants. All patients were age 45 years or older. Eighty age-matched apparently healthy men without CaP or benign prostatic hyperplasia were recruited as controls. Patients with CaP were divided into two subgroups: those who had received no treatment (treatment-naïve patients with CaP) and those who had received ADT (ADT-treated patients with CaP). Treatment modalities included use of surgical orchiectomy and medical castration (GnRH, biclutamide, and flutamide). The treated CaP subgroup was further subdivided between those who had received treatment for up to 1 year and those who had received treatment for more than 1 year. The treated CaP subgroup was again divided into two categories according to treatment type: patients treated with orchiectomy and patients treated with hormone manipulation alone.

This study was carried out in accordance with the World Medical Association Declaration of Helsinki.^15^ The purpose and nature of the research were explained to each participant, and informed consent was obtained. A standard questionnaire was administered to participants to obtain information about their age, family history, and dietary and physical lifestyles.

### Sample Calculation

The number of samples in this research was determined using the Kish^16^ formula:

$$\text{N}=\frac{{\text{Zα}}^{\text{2}}\text{pq}}{{\text{d}}^{\text{2}}}$$

where N = the desired sample size, Zα = the α level of the coefficient interval at 95% (1.96), p = the proportion of nonoccurrence, q = (1 − p) proportion of nonoccurrence, and d = precision.

Substituting the expected occurrence of p = 11% (ie, 0.11), we have

$$\begin{array}{c} \text{N}=\frac{1.96^{2}\times 0.11\left(1-0.11\right)}{{\left(0.05\right)}^{2}}\\ =150.4 \end{array}$$

### Sample Collection

Blood samples were taken from each participant between 8 and 10am after an overnight fast and 3 days of abstinence from sexual activity; 10 mL of blood was aseptically collected into plain bottles and left to clot, and the serum was extracted and stored frozen until used.

### Measurement of Physical Parameters

Weight and height of each participant were measured. Measurement of weight in kilograms was performed using a weighing balance, and measurement of height in meters was performed using a stadiometer. Waist circumference in centimeters was calculated by taking the average of two measurements, one after breathing in and the other taken after breathing out, at the midpoint between the top of the iliac crest and the bottom of the rib cage using an inelastic calibrated tape. Body mass index (BMI) was calculated by dividing the weight in kilograms measured to the square of height in meters of each participant.

### Assay Methods

Prostate-specific antigen (PSA) was determined with an enzyme-linked immunosorbent assay kit obtained from Syntron Bioresearch (Carlsbad, CA). Insulin was determined with an enzyme-linked immunosorbent assay kit obtained from DRG Instruments (Marburg, Germany). Glucose, total and HDL cholesterol, and triglyceride levels were determined using the enzymatic colorimetric methods with kits obtained from Giesse Diagnostics (Rome, Italy). Friedewald’s equation was used to calculate both VLDL and LDL cholesterol levels. All tests were carried out according to the instructions of the manufacturers.

### Statistical Analysis

Statistical analysis was performed using PAWstatistic 18, a statistical package from SPSS (Chicago, IL). Results are expressed as mean plus or minus standard deviation. *t* test and analysis of variance were used to analyze the data. Post hoc analysis was performed using least significant difference. The level of significance was set at 95% CI, where a probability value of *P* less than .05 was regarded as statistically significant.

## RESULTS

A comparison of PSA level, lipid profile, indices of central obesity, glucose level, and insulin level in treatment-naïve patients with CaP and ADT-treated patients with CaP and controls showed significant variations in mean values of PSA (*P* < .001), total cholesterol (*P* = .016), waist circumference (*P* = .050), and BMI (*P* = .003) among the groups. However, no significant variations were observed in mean glucose or insulin levels among groups (Table 1).

**Table 1:**
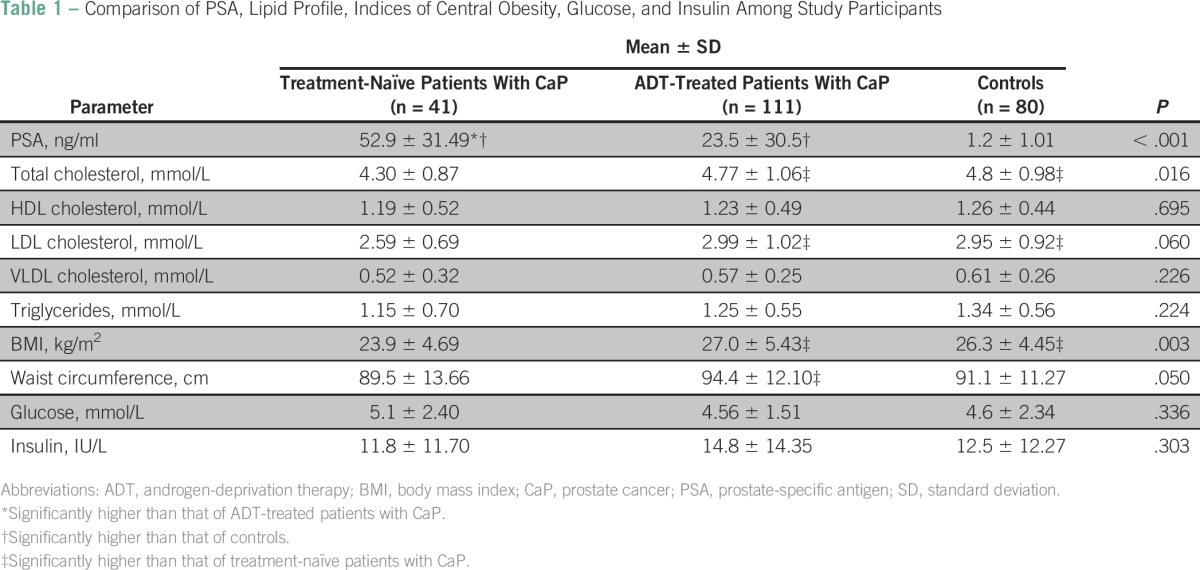
Comparison of PSA, Lipid Profile, Indices of Central Obesity, Glucose, and Insulin Among Study Participants

Post hoc analysis showed that the mean values of total cholesterol (*P* = .010), LDL cholesterol (*P* = .021), waist circumference (*P* = .029), and BMI (*P* =.001) were significantly higher in the ADT-treated group compared with the treatment-naïve group. All these parameters were comparable between the ADT-treated group and controls. The treatment-naïve group had significantly lower mean values of total cholesterol (*P* = .007), LDL cholesterol (*P* = .049), and BMI (*P* = .013) than the controls (Table 2).

**Table 2:**
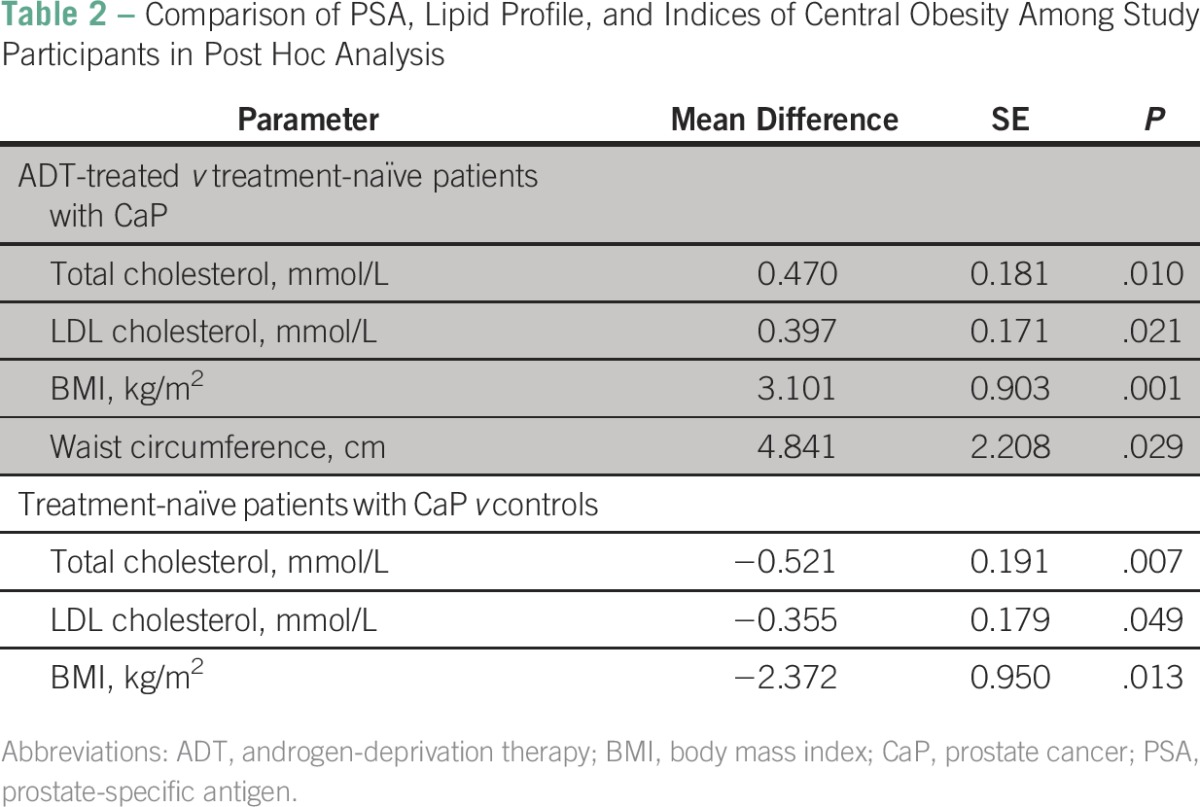
Comparison of PSA, Lipid Profile, and Indices of Central Obesity Among Study Participants in Post Hoc Analysis

A comparison of PSA level, lipid profile, indices of central obesity, glucose level, and insulin level in treatment-naïve patients with CaP, patients treated with ADT for up to 1 year, and patients treated with ADT for more than 1 year showed significant variations in mean values of PSA (*P* < .001), LDL cholesterol (*P* = .043), and BMI (*P* = .003). Insulin and glucose levels were comparable in the three groups (Table 3).

**Table 3:**
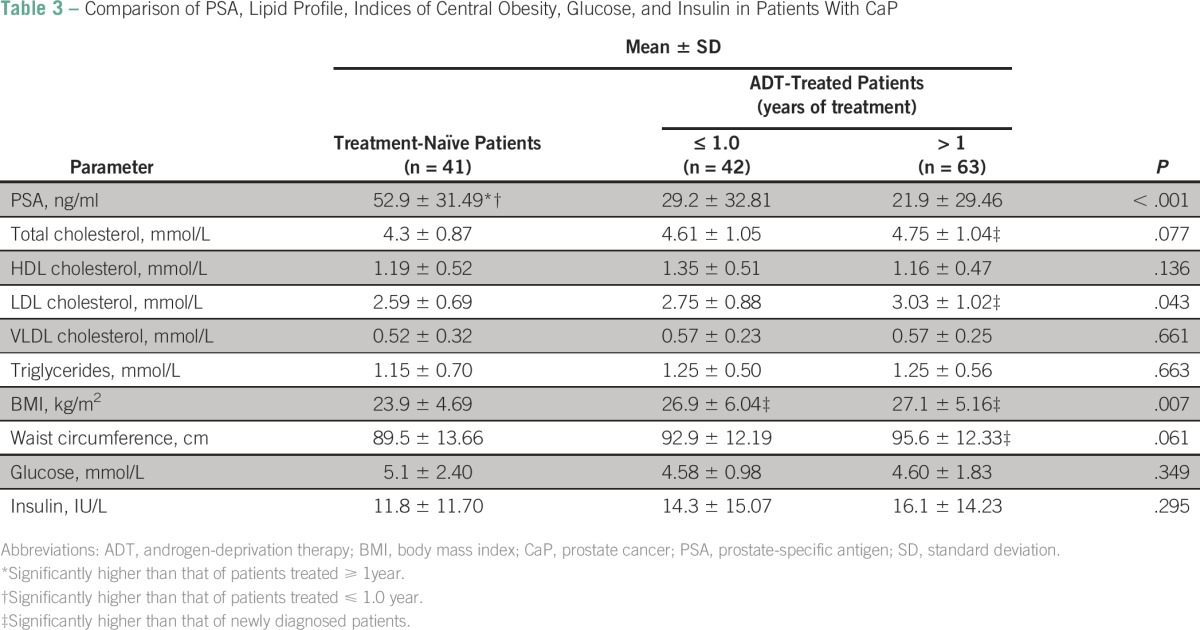
Comparison of PSA, Lipid Profile, Indices of Central Obesity, Glucose, and Insulin in Patients With CaP

In patients treated with ADT for up to 1 year, only BMI was significantly higher compared with treatment-naïve patients. Those treated for more than 1 year had significantly higher BMI, waist circumference, total cholesterol, and LDL cholesterol values when compared with treatment-naïve patients with CaP (Table 4).

**Table 4:**
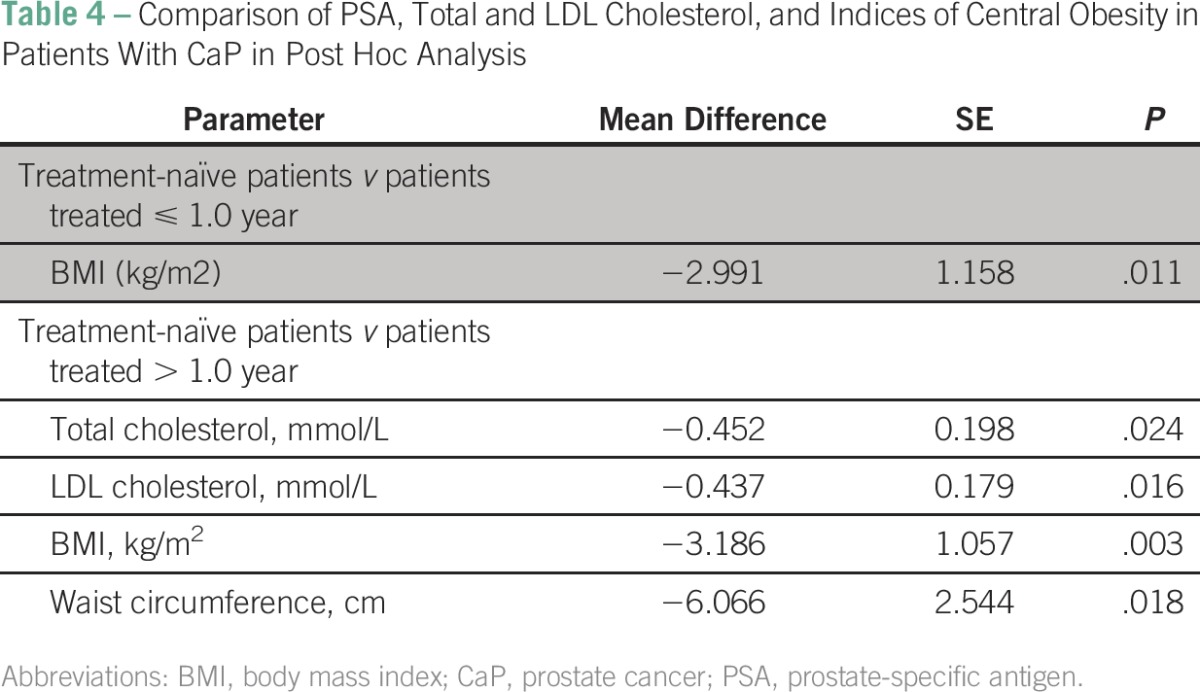
Comparison of PSA, Total and LDL Cholesterol, and Indices of Central Obesity in Patients With CaP in Post Hoc Analysis

A comparison of PSA level, lipid profile, indices of central obesity, glucose level, and insulin level in treatment-naïve patients with CaP, patients treated with hormone manipulation alone, and patients treated with orchiectomy showed significant variations in mean values of PSA (*P* < .001), total cholesterol (*P* = .025), and BMI (*P* = .002). Those treated with orchiectomy had significantly higher PSA (*P* < .001) and waist circumference values only. Of patients receiving treatment, 40 (36%) chose orchiectomy as a treatment option, whereas 71(64%) chose hormonal manipulation alone (Table 5).

**Table 5:**
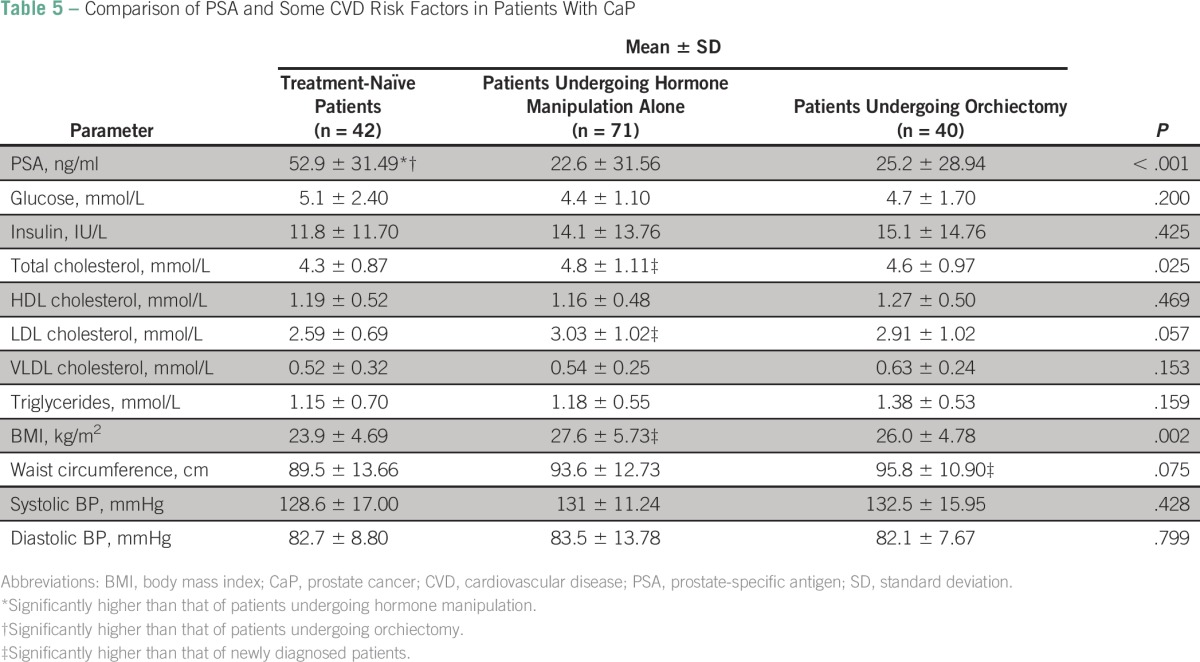
Comparison of PSA and Some CVD Risk Factors in Patients With CaP

The group receiving hormone manipulation alone had significantly higher mean values of PSA (*P* < .001), total cholesterol (*P* = .007), LDL cholesterol (*P* = .017), and BMI (*P* < .001) when compared with the treatment-naïve group as shown by post hoc analysis. However, mean glucose and insulin levels showed no significant variations (*P* = .027) among the groups (Table 6).

**Table 6:**
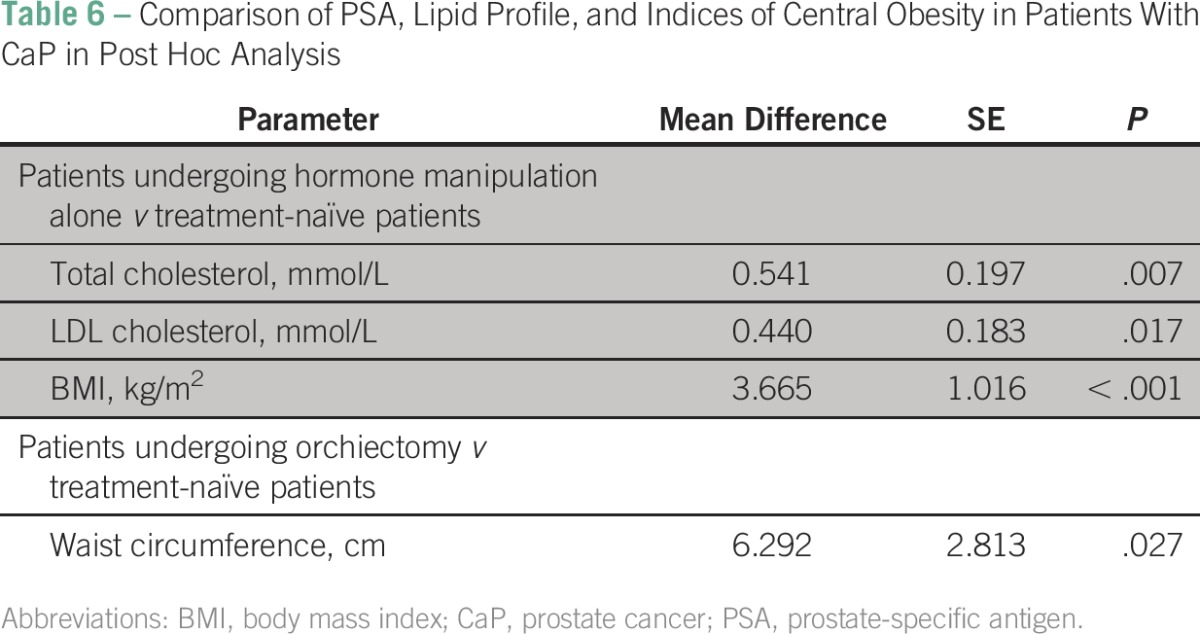
Comparison of PSA, Lipid Profile, and Indices of Central Obesity in Patients With CaP in Post Hoc Analysis

## DISCUSSION

The patients who received ADT in our study had significantly higher total cholesterol and LDL cholesterol levels than the treatment-naïve patients, but these levels were comparable to those of the controls. These changes in lipid profile may have resulted from ensuing hypogonadism after ADT. The beneficial effects of testosterone are considered to occur via its aromatization to estrogen.^17^ To further support this, it has been shown that administration of anabolic agents, which are nonaromatizable, increases LDL cholesterol and decreases HDL cholesterol levels.^18^ Anabolic agents achieve this by increasing hepatic lipase activity, which enhances the catabolism of HDL. In contrast, hepatic lipase activity is only slightly increased with testosterone administration.^19^ The pattern of changes in lipid profile observed in our study was also reported in a study by Braga-Basaria et al,^17^ who reported that men receiving long-term ADT had significantly higher fasting levels of total and LDL cholesterol than patients not receiving ADT. However, their ADT group also had higher total and LDL cholesterol levels compared with the control group. A similar observation was also made by Torimoto et al^13^ in a prospective study of Japanese men receiving ADT. However, in another prospective study by Dockery et al,^20^ men receiving ADT had a significant increase in both total and HDL cholesterol levels after 3 months of treatment, with no change in either LDL cholesterol or triglyceride levels. ADT for 6 months in patients with CaP resulted in elevated total cholesterol but no change in HDL cholesterol or triglyceride levels. Similar findings were observed by Smith et al^21^ and Nishiyama et al.^22^ Smith et al^23^ reported that ADT for CaP using GnRH agonist treatment of a period of months resulted in significantly increased triglyceride levels. Chen et al^24^ reported that ADT for 2.5 years resulted in significantly increased triglyceride and decreased HDL cholesterol values; however, only HDLC3 was affected. Another prospective study of 40 men who received ADT for a year showed a 9% increase in total cholesterol, 7.3% in LDL cholesterol, and 26.5% in triglyceride levels.^25^ However, there was also an 11.3% increase in HDL cholesterol levels. These observations are not universal, because one prospective study reported that there was no change in any of the lipids after patients had received 3 months of ADT.^21^ In our study, we did not observe any significant variations in HDL cholesterol or triglyceride levels. The differences in the reported lipid profiles may have resulted from variations in study design. Factors that may have contributed to the variations include method of ADT, therapy duration, and sample size. Regardless of this, most of the studies observed an enhancement of unfavorable lipid profiles in response to ADT.

Patients who were treated with ADT had higher mean values of BMI and waist circumference compared with treatment-naïve patients. This increase in measures of central obesity was one of the earliest metabolic changes observed after ADT, with significantly higher BMI observed in those receiving ADT for up to 1 year and higher BMI and waist circumference in those treated with ADT for more than 1 year. In a study by Fox et al,^26^ approximately 39% of patients with CaP had cachexia. One of its prominent clinical features is weight loss and increased muscle protein breakdown. It can occur in patients with metastatic or localized disease.^27^ Although this could be the reason for the lower BMI of treatment-naïve patients, our study did not examine patients for cachexia. The higher BMI in treated patients may have been an effect of treatment, which is associated with weight gain. Although the mechanisms are not well understood, it has been reported that visceral adipocytes have androgen receptors and testosterone may be involved directly in free fatty acid mobilization. Therefore, any deficiency in testosterone will result in diminished lipolysis in the adipocytes and thus enhance the buildup of fat stores in the abdomen.^13,28^ This increasing adiposity may begin the cascade of events leading to metabolic complications. These changes may ultimately predispose patients to CVD.^29^ Similar observations were made by Chen et al,^30^ who reported a significant reduction in lean body mass and increase in total body fat mass in patients with CaP after 1 to 5 years of ADT. Smith et al^23,25^ also observed increases in BMI, fat mass, and total body weight after ADT when compared with baseline. In a study by Cleffi et al,^14^ patients receiving ADT had a greater waist circumference compared with those not receiving ADT. However, BMI was similar in both groups, supporting the occurrence of visceral obesity in the ADT group in their study. In a prospective study by Torimoto et al,^13^ ADT resulted in an increase in fat mass, particularly visceral fat. They also reported a negative correlation between testosterone concentrations and the degree of central abdominal obesity in the healthy male population.^13^

In our study, levels of fasting insulin and glucose were comparable between patients with CaP and controls. This was different from observations made by Smith et al^23^ and Basaria et al,^31^ who reported a greater risk of development of insulin resistance and hyperglycemia in men receiving ADT. Basaria et al, after adjustment for age and BMI, observed significantly higher fasting levels of insulin and glucose in men receiving ADT. In a 3-month prospective study of 25 nondiabetic patients with CaP receiving ADT, Smith et al reported a 12.8% decrease in mean insulin sensitivity from baseline and a concomitant 25.9% increase in fasting plasma insulin levels. Shahani et al^29^ reported that short-term ADT (within 3 to 6 months) resulted in early metabolic changes that led to hyperinsulinemia, whereas long-term ADT (≤ 12 months) was associated with a higher prevalence of metabolic syndrome and diabetes when compared with controls.

Other studies have found higher levels of insulin in patients with CaP, patients with lethal CaP, and patients with poorly differentiated CaP than in controls and patients in other situations.^12,32,33^ It is of note that these studies were carried out in white populations, whereas our study involved African men. In a study by Cleffi et al^14^ of Hispanic men, the mean glucose levels of patients with CaP receiving ADT and those with CaP not receiving ADT were comparable.

In our study, change in body composition occurred as early as 1 year after ADT initiation. There was a significant increase in BMI and waist circumference values when compared with those of treatment-naïve patients. Those who had been receiving ADT for more than a year had significantly higher mean values of BMI, waist circumference, total cholesterol, and LDL cholesterol when compared with treatment-naïve patients. These characteristics are all strong CVD risk factors. It can therefore be inferred that treatment with ADT significantly increases the risk of this group of patients experiencing CVD events. Studies by various authors have all shown that in men treated with ADT, CVD events and myocardial infarction rates are high.^21,34-41^ However, hyperinsulinemia and hyperglycemia after ADT, which have been reported in other studies, were not observed in our study. The pattern of dyslipidemia in our study was related mostly to increases in total cholesterol and LDL cholesterol levels. There was no hypertriglyceridemia, which was observed in some other studies in whites.^11,21,29,31^ The reason for this may be the differences in population types. Those studies were carried out among whites and ours was African based.

The fact that patients who underwent orchiectomy in our study had fewer CVD risk factors compared with their counterparts who underwent hormonal manipulation alone suggests that orchiectomy may be a better option for ADT. This is supported by observations by Torimoto et al,^13^ who reported that patients undergoing hormone manipulation alone were observed to have higher total and LDL cholesterol levels, greater waist circumference, and increased body weight after 12 months of treatment.^13^ The only problem with this option is that it may be unpopular with men in Africa, where virility is seen as the greatest essence of manhood; only 36% of patients in our study chose orchiectomy as an ADT option, compared with the 64% who opted for hormonal manipulation alone.

In conclusion, this study shows that ADT results in elevated total cholesterol, LDL cholesterol, BMI, and waist circumference values, which are all strong risk factors of CVD. Therefore, screening for these risk factors should be integrated into treatment plans for these patients.
